# Design and implementation of a user office system for the Canadian Light Source

**DOI:** 10.1107/S1600577525000153

**Published:** 2025-02-06

**Authors:** Kathryn Janzen, Michel Fodje

**Affiliations:** a Canadian Light Source 44 Innovation Boulevard Saskatoon SKS7N 2V3 Canada; University of Manchester, United Kingdom

**Keywords:** *USO* software, user office software, synchrotron facilities, user management, Canadian Light Source

## Abstract

To streamline the management of thousands of users and proposals for synchrotron experiments at the Canadian Light Source (CLS), we developed a modern, web-based user services software system that enables the facility to transition away from manual paper-based processes. This user-friendly system offers comprehensive features for managing users, proposals, reviews, sample safety and publications. It has been in active use at the CLS for six years and has simplified and significantly transformed user operations. The system is designed for integration into other large-scale facilities with minimal effort, and the source code is publicly available.

## Abstract 2

An open-source software system for managing user office operations at a large-scale research facility is presented.

## 1. Introduction

Large-scale scientific facilities such as the Canadian Light Source (CLS) and other synchrotron and neutron facilities support the research of many national and international research groups from academic, governmental and industrial institutions. Each year, these facilities serve thousands of projects, each involving multiple team members and samples with different experimental and safety requirements at one or more beamlines or instruments within the organization (Simoulin, 2017 ▸; Hallonsten, 2013 ▸; Söderström *et al.*, 2022 ▸).

Therefore, efficient management of processes around user operations is a crucial component determining the success of these large-scale facilities. These processes include user registration, proposal submission, technical and scientific review, beam time allocation, scheduling, safety review, access control, research output and publications tracking, user feedback, and reporting, among others. Each of these functions often requires the participation of multiple personnel from across the organization.

Traditionally, many facilities including the CLS started with a manual approach where, although computers were used to prepare and exchange documents and spreadsheets, most of the business logic required to implement user management was accomplished by administrative procedures, human-to-human interaction and multiple unstructured manual steps. This approach was not sustainable, motivating the development of digital user office software systems at many large-scale facilities. Despite the widespread need for such software systems, detailed descriptions of their design and implementation have been scarce in the literature. The only example known to the authors is the *Digital User Office* (*DUO*) software developed at the Paul Scherrer Institute in Switzerland and used by multiple large-scale European facilities. *DUO* is based on an Oracle database back-end and a web-based front-end written in PHP, with direct interaction between the PHP front-end and the database using SQL commands (Bertrand & Weyer, 2005 ▸). Although highly successful, as shown by its widespread use, the *DUO* source code is not publicly available. The proprietary *Virtual User Office* (*VUO*) software developed at the ELETTRA synchrotron facility in 1998 has been briefly mentioned in the literature (Billè *et al.*, 2015 ▸), and a more recent system, the *SOLARIS DUO*, written in Java, has also been briefly described (Szymocha *et al.*, 2017 ▸).

This paper describes the design and implementation of *User Services Online* (*USO*), an open-source web-based software system that leverages modern technology and web standards to implement all the major features required to manage user operations at a large-scale facility. The description includes design principles, key functionalities, implementation details and its impact on facility operations at the CLS.

## 2. Implementation details

### 2.1. System architecture

**Figure 1:**
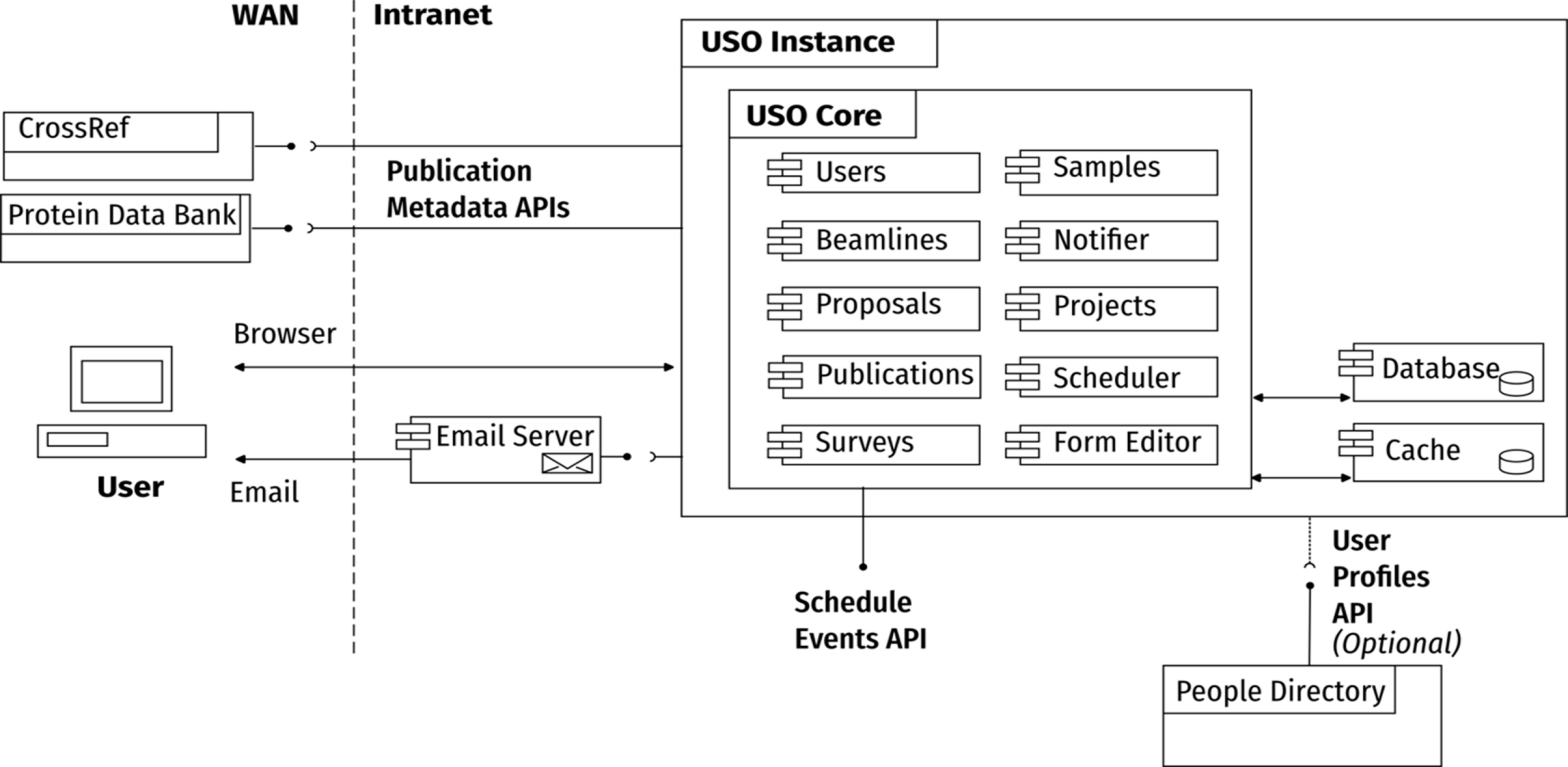
System architecture diagram for the *USO* system. The core *USO* application modules are shown in the inner block. Interactions with other data sources and users are illustrated together with provided APIs.

Fig. 1 ▸ illustrates the system’s software architecture. The main *USO* application consists of a back-end that implements all the business logic and interacts with a relational database, and a front-end that provides user interfaces and application programming interfaces (APIs) for interacting with the system. Additionally, the system can optionally rely on other services for authentication and fetching roles, permissions, and user profile details.

### 2.2. Technology stack

The *USO* is developed using Python and the Django web application framework (Chen *et al.*, 2020 ▸). The Django framework was chosen for its excellent abstraction of interactions with relational databases in the Django object-relational mapper (ORM) and the intuitiveness of its model–view–controller design pattern. PostgreSQL was chosen as the relational database due to the maturity of its support within Django. The Django ORM optimizes database access by combining lazy loading, query caching, indexing, transaction management and efficient SQL (structured query language) generation. It converts queries and database operations written in high-level Python code to optimized and efficient SQL, minimizing database requests while supporting features like aggregation and annotations for high-performance database-side computations (Chen *et al.*, 2020 ▸). As a result, the *USO* system maintains high performance and responsiveness even as the number of database records grows. For example, no noticeable performance degradation was observed with 20000 user entries in the database during testing.

The front-end interfaces are developed using standard web technologies such as HTML5, CSS3 and JavaScript. The *USO* system is deployed on an Apache web server through the Web Server Gateway Interface (WSGI) running within a Docker container. Alternatively, it can be deployed on any server supporting the WSGI standard. The source code consists of approximately 19000 lines of Python, 10500 lines of HTML, 5500 lines of CSS and 1500 lines of JavaScript.

### 2.3. Development methodology

The software was developed following the Agile methodology, with core features developed as individual Django applications. A usable product was available for testing throughout the development process as features were added progressively. This allowed stakeholders early access to test the system and provide feedback to the developers. The developers met regularly with key stakeholders such as user office staff, industrial science staff, health and safety staff, and beamline/instrument scientists to discuss design choices and perform usability testing throughout the development process. A subset of the core application modules developed, listed in order of development, are *Users*, *Publications*, *Form Editor*, *Beamlines*, *Proposals*, *Projects* and *Scheduler*.

### 2.4. User interface design

A key design objective was to produce a modern, responsive, user-friendly interface for all system users, including researchers, staff and reviewers. Consistent with this approach, we aimed to minimize the information requested of users through forms and prompts. We also relied on the progressive disclosure design principle to reveal functionality and features based on the user’s role or permissions.

**Figure 2:**
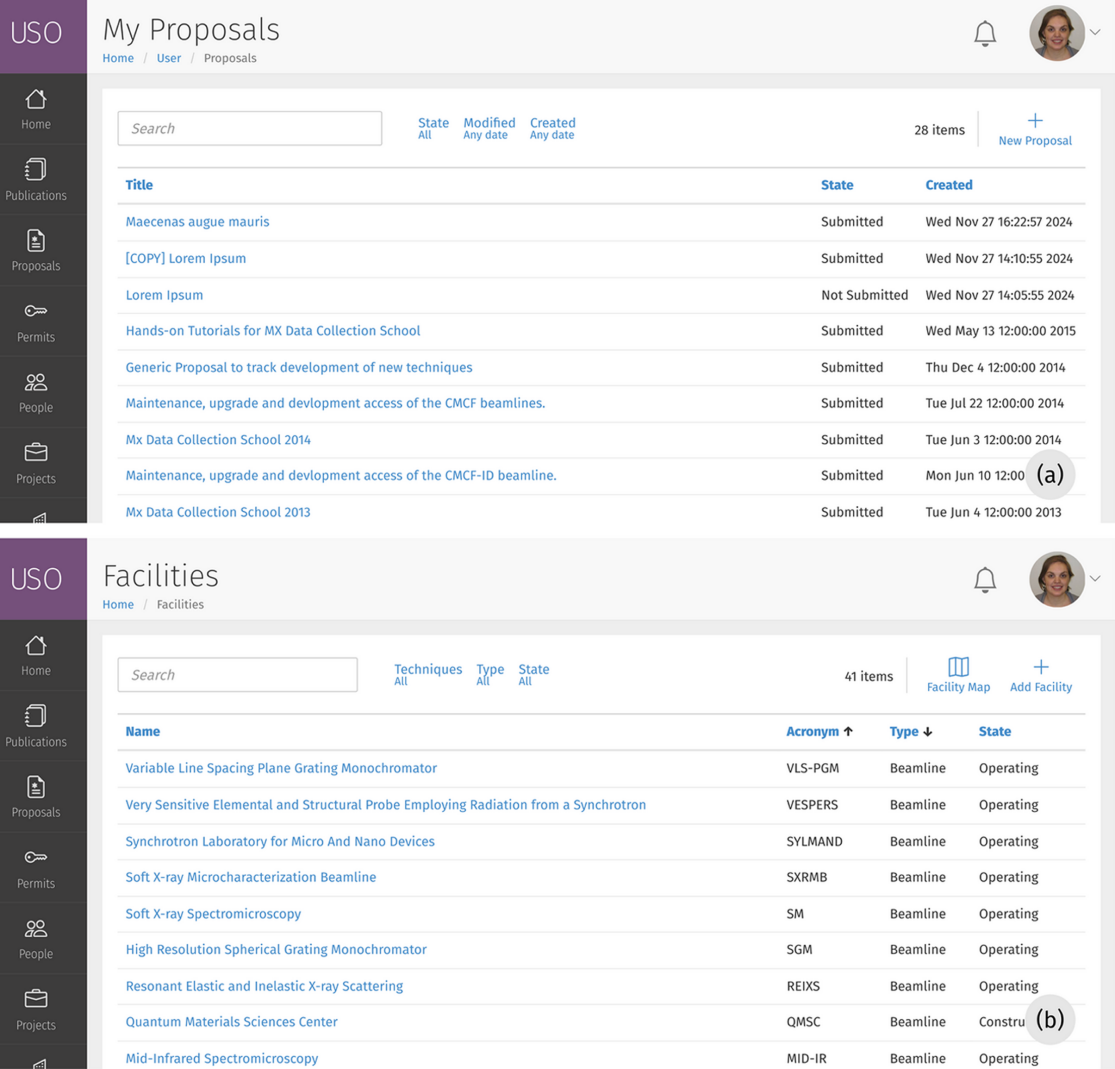
Screenshots of examples of the ‘list view’ user interface. Every list view includes a search bar and a configurable selection of filters to allow searching for items. Links are sometimes used to redirect users to a detail page for the selected item or to display a form overlay for editing the item. Icons at the top of the list also provide additional tools, *e.g.* for adding new entries.

We employed a limited set of interface metaphors carefully designed with reusable templates to provide a consistent user interface. These include list views, detail pages, forms and dashboard tiles. Examples of these metaphors are shown in Figs. 2 ▸, 4(*b*) and 6. Almost every aspect of the user interface is based on one of these elements, enabling users to quickly learn how to interact with the system (Neale & Carroll, 1997 ▸).

## 3. Key functionalities

The *USO* software system supports many features centred around user interactions with a large-scale facility like the CLS and activities routinely performed by facility staff to support users. In summary, the following feature sets are currently implemented in the system:


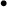
 Management of users: registration, user profiles, institutions and agreements.


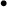
 Beamline/instrument management: configuration of available techniques.


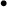
 Proposal creation and editing: management of sample lists and hazards.


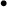
 Scientific, technical, safety, ethics and equipment reviews; review tracks with different review committees; reviewer profiles with subject areas and techniques.


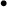
 Publications management: submission of journal articles, theses and patents; tracking of Protein Data Bank depositions; automatic citations tracking; metrics reporting.


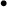
 Allocation of shifts to approved projects: scheduling of beam time and user support.


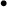
 Management of projects and research teams: beam time requests, samples and other experimental materials, beamline and laboratory sessions for academic and industrial users.


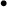
 Generation of statistics, metrics and reporting


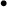
 User feedback.

A few of these features are described in more detail below.

### 3.1. User management and security

**Figure 3:**
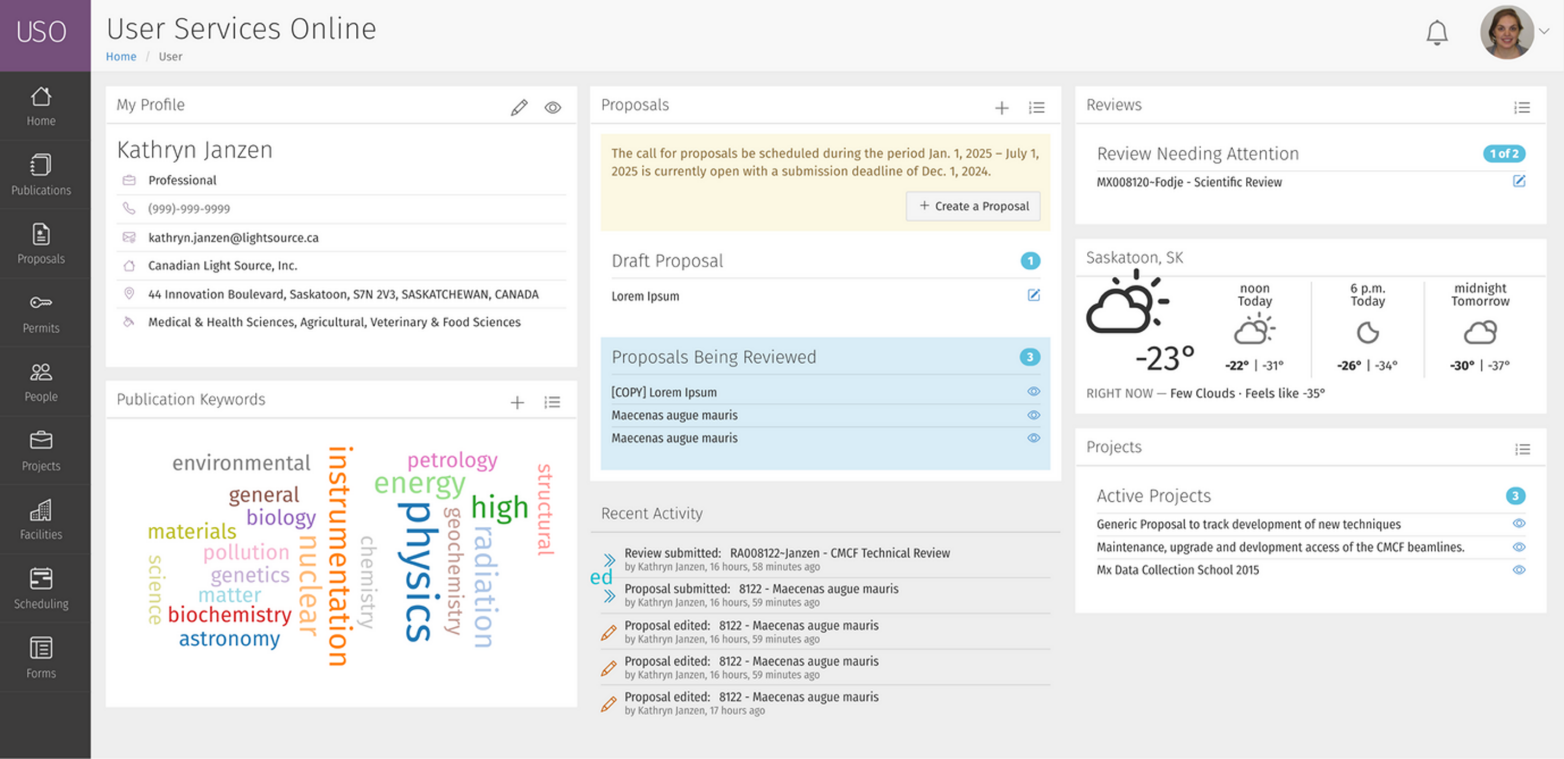
Screenshot of the *USO* dashboard view for a user with administrator roles. The menu system on the left is dynamic and changes based on the user’s roles. Each menu item expands to reveal sub-menus for access to additional functionality. Panels are used to summarize relevant and essential information.

Access to the system is controlled through user accounts, which new users can request through a registration form. After successful registration and verification of the user’s email address, an account is created, allowing new users to log on to the system. Logged-in users have a dashboard that provides an overview and menus for accessing system functions, as shown in Fig. 3 ▸.

The software includes the capability of synchronizing user profiles with an external source through an API, allowing existing external accounts to log in or create new accounts in an external system after registration. Each user within the system can have one or more ‘roles’ and ‘permissions’ assigned to them. A *USO* role is a prescribed function or status conferred on a person, and a *USO* permission is a qualification that allows a person to perform a task, irrespective of their role. Examples of roles include *User*, *Reviewer* and *Beamline Staff*; and examples of permissions include *Facility Access*, *Laboratory Access*, *Animal Work* and *Cryogenic Work*.

The *USO* system uses roles and permissions to control page access and select which menu or navigation items are shown to an authenticated user. For example, the ability to edit beamline/instrument specifications and techniques is only available to the administrator role for the specific beamline/instrument and user interface elements for performing those actions are hidden from other users. This prevents users from seeing menus to pages they cannot access, simplifying the interface. The roles-based access system provides a powerful mechanism for implementing all the controls required for a complex system where different individuals from across the organization must interact with various aspects of the system.

### 3.2. Proposal management

Within the *USO* system, a proposal is a document submitted by a prospective user describing planned research, the samples and other materials they will use, the facilities or instruments being requested, and the techniques they plan to use at those facilities. To facilitate this process, the *USO* system allows beamline or instrument scientists to configure their facilities to specify available techniques, parameters and instrument availability during research cycles (6 month blocks).

**Figure 4:**
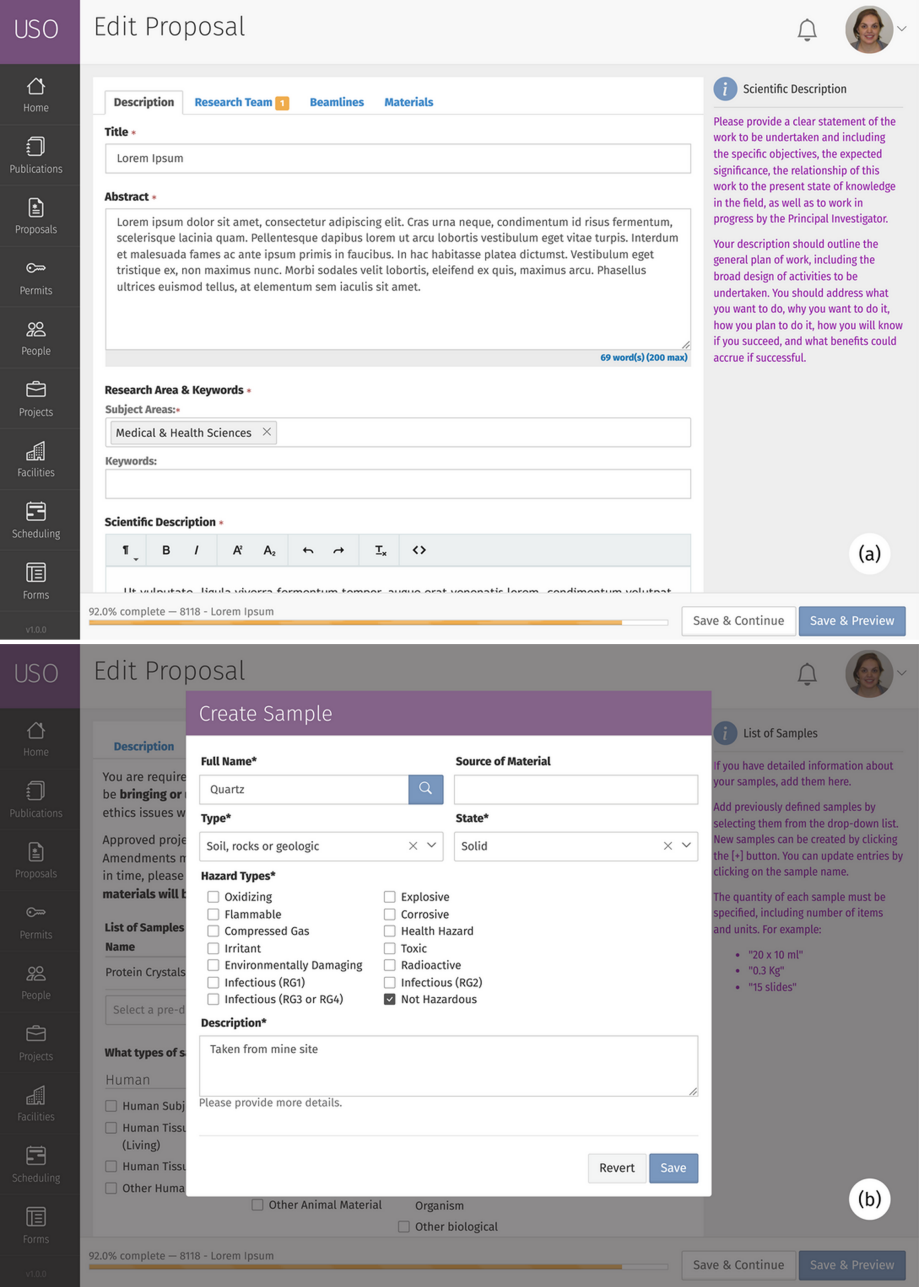
Screenshots of (*a*) the proposal form and (*b*) the sample form. The sample form is launched as an overlay pop-up window within the proposal form when users need to add new samples to their proposal. Similar forms are used to edit different items within the interface.

*USO* provides user-friendly forms allowing research proposals to be created, edited and submitted for review. The proposal form considers the previously configured beamline/instrument availability when displaying choices for users. It also contains relevant built-in help about the capabilities of each beamline/instrument based on configured beamline parameters. Additionally, users can add samples to their proposals and edit sample properties, including hazard information – specific GHS hazards can be associated with each sample for use during the safety review process. Added samples are saved in a personal samples database for reuse later in other proposals. Fig. 4 ▸ illustrates some of these features.

Completed proposals can be submitted to one of many configurable review tracks. The availability of review tracks at submission depends on the user’s roles, the selected access mode (for example, Staff, Priority, Purchased, Education or General), the status of the requested cycle (whether a call-for-proposals is open or not), and the beamlines or instruments requested in the proposal. This submission scheme provides flexibility to support a variety of proposal types.

### 3.3. Management of cycles, review tracks and reviews

The *USO* system manages research time in cycles. A cycle is a period, typically 6 months long, during which experiments are scheduled and performed at the facility. Therefore, calls for proposals and reviews are always performed relative to a specific cycle. However, the dates for opening/closing the calls can be several months before the start of the cycle to allow enough time for reviews, scheduling and notification. Therefore, the system enables user office staff to configure each cycle, specifying when calls for proposals should be open and closed and giving due dates for beamline/instrument configuration, reviews, shift allocations and scheduling.

A ‘review track’ in the *USO* system is a prescribed sequence of reviews to which proposal submissions are subjected before approval. The *USO* supports the definition of multiple review tracks, each with a separate committee of reviewers. During beamline/instrument configuration, individual techniques can be assigned to specific review tracks, ultimately determining how the submissions containing those techniques will be reviewed. A special internal review track is used for industry or purchased access proposals.

**Figure 5:**
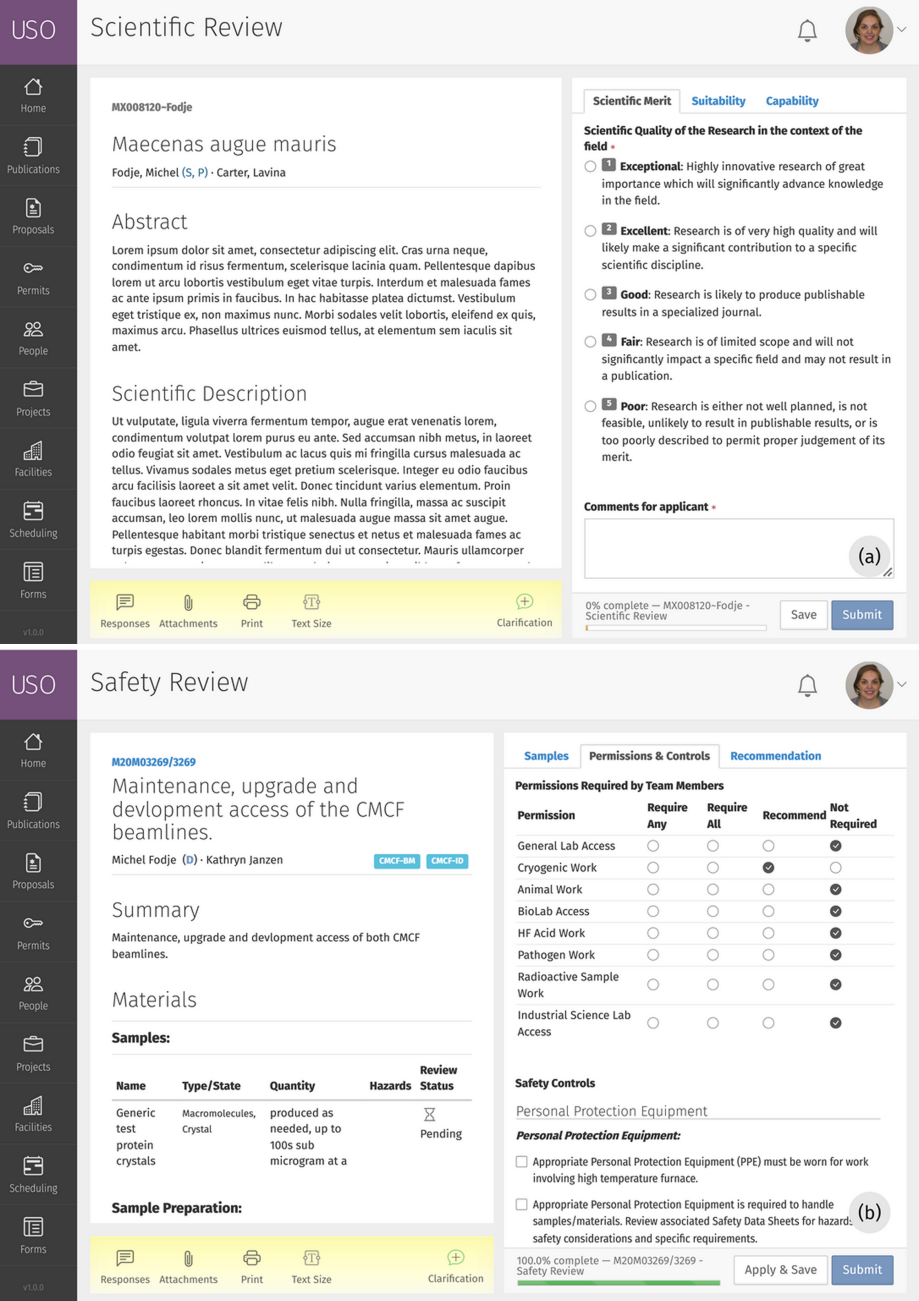
Screenshots of the (*a*) scientific review and (*b*) safety review forms. The formatted proposal is shown on the left, and the review form is shown on the right. Reviewers can request or view clarifications and download PDF versions of the proposal using the tools on the bottom left.

On submission, the *USO* system assigns each proposal a technical review to be completed by the beamline/instrument scientist. Additional reviews are manually or automatically assigned to reviewers with areas of expertise that match the research topics specified in the proposal. Automatic reviewer assignment uses a constraint-based optimization algorithm (Karimzadehgan & Zhai, 2009 ▸). Reviewers complete and score assigned reviews using a user-friendly online form, as shown in Fig. 5 ▸. Once reviews are complete, the *USO* software provides additional features to support peer-review committee meetings and the allocation of beam time to approved proposals. For example, each beamline/instrument can specify the proportion of beam time reserved for academic, industry or other access categories to facilitate allocating shifts to proposals within that category. The system then uses the review scores to suggest cut-off points based on the ranking of proposals, the requested number of shifts and the available shifts within the category. A feature for reserving shifts in anticipation of future needs is also provided. This allows the system to support industrial science projects that arise outside of the regular allocation calendar.

### 3.4. Managing projects and scheduling

**Figure 6:**
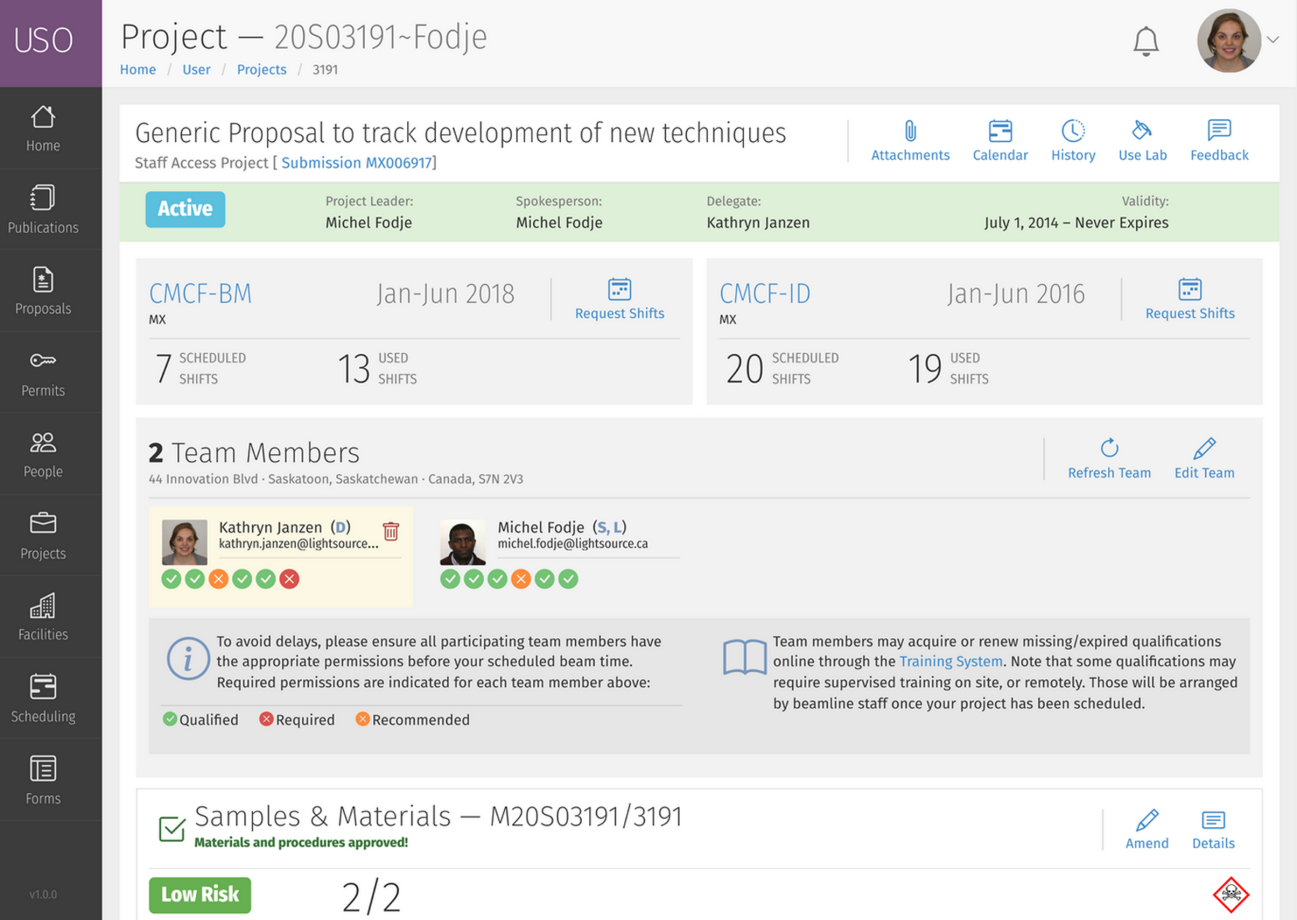
Screenshot of a project page. Tools for managing the project are shown at the top. Users can manage project team members, reserve laboratory space and view their beam time schedule.

At the end of allocation, projects are automatically created for all proposals that successfully pass the technical and scientific review process. Projects can support multiple beamlines and different amounts of allocated beam time for each beamline. The duration of the project’s validity varies based on the requested access mode and the type of review track through which the proposal was reviewed. Projects can be reallocated beam time on request for additional cycles throughout their validity period. Extra features available include viewing the review report for the proposal, managing research team members, updating samples and materials, requesting beam time, signing on to beam time sessions, providing user feedback, reserving ancillary laboratories, and viewing and replying to safety review clarification requests. An example project page is shown in Fig. 6 ▸.

The *USO* system includes a scheduler that allows beamline/instrument scientists to schedule projects for each cycle based on a master facility schedule overlay prepared for the cycle by user office staff. The overlay schedule designates shifts available for beam use, machine maintenance and development. The master schedule is also the source of shift counts used to determine the total number of available shifts during beam time allocation. Furthermore, when creating proposals, users can specify scheduling preferences, and if provided, these preferences are displayed within the scheduler to assist beamline/instrument staff in preparing the final schedule. Additionally, beamline/instrument scientists can assign user-support shifts to staff within the schedule.

### 3.5. Safety review and permitting

A safety review is performed on the specified experimental plan and the samples and materials within the project. Though safety reviews are accomplished similarly to technical and scientific reviews, they are done at the project stage rather than at the proposal stage. During a safety review, health and safety staff can recommend or require any number of permissions to the project based on the hazards associated with the work being performed, with the flexibility to require permissions for any or all team members. Team members participating in the session must have these permissions.

Additionally, beamline/instrument scientists must approve each session before it can commence. A research team representative initiates the session by selecting all the samples to be measured and the team members participating during the experiment. The *USO* system then verifies that all the selected samples have been approved and the selected team members have the required permissions before creating a valid permit for the experiment to commence. The session permits are valid for the duration of the experiment as specified in the schedule. However, users or beamline staff can terminate the session earlier, and beamline staff can extend the duration beyond the scheduled end time if needed.

### 3.6. Publication management and reporting

A critical need of large-scale research facilities is the ability to track research output resulting from research performed at the facility (Hallonsten, 2013 ▸), such as the numbers of peer-reviewed publications, theses, patents, conference proceedings and depositions in global repositories like the Protein Data Bank (PDB). To satisfy this need, the *USO* system allows users to submit publications and theses for review and addition to the facility publications database. Submission is often as easy as providing a DOI, patent number or ISBN. The system fetches additional metadata from online databases such as CrossRef (Hendricks *et al.*, 2020 ▸) and Google Patent Search. Furthermore, the system periodically fetches citation metrics for each article within the database. In the case of PDB depositions, entries associated with the facility and their corresponding peer-reviewed articles are fetched automatically without any user intervention using the public PDB APIs. Publications submitted by users are triaged by staff to ensure database integrity and are assigned to specific beamlines/instruments. Users can also claim submitted publications to associate them with their accounts. This allows beamline staff to use the publication track record of research team members when scoring the team’s capability during the technical review of future proposals.

**Figure 7:**
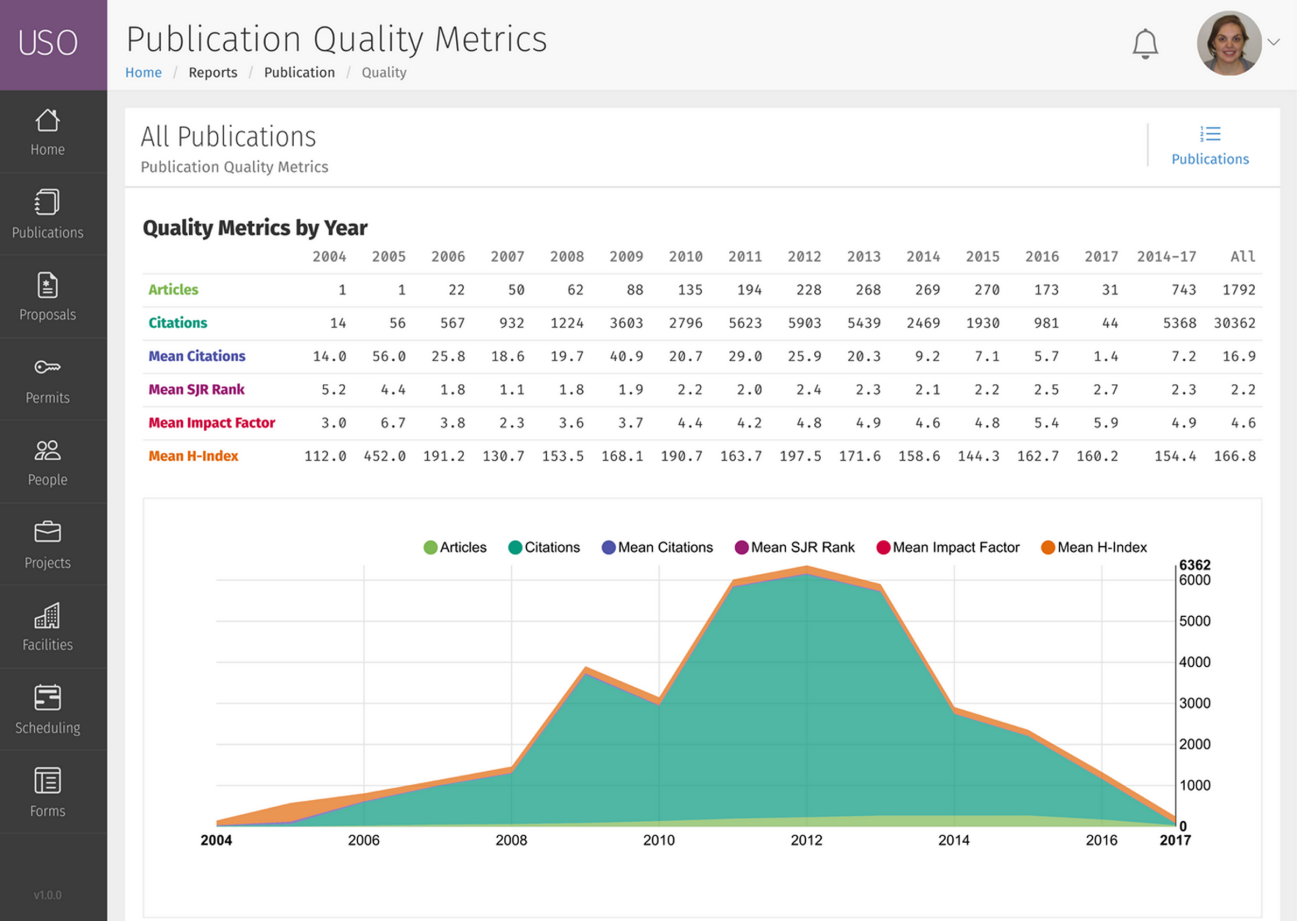
Screenshot of facility publication metrics, showing tables and responsive graphs. Similar reports are available for each sub-unit or beamline within the facility.

Based on the wealth of information in the publications database, the system generates publication reports for the whole facility and individual beamline/instrument groups (see Fig. 7 ▸).

### 3.7. Communication and notifications

Communication is an essential aspect of the *USO* system. Notifications are used extensively within the interface to provide feedback about system actions. Additionally, email messages are used to notify users when offline. Some examples of contexts in which email messaging is used include confirmation of registration and account creation, confirmation of submission of proposal, reviewer notification of pending reviews needing their attention, notification of requests for clarification to users, approval or rejection of proposals and allocation of beam time, and reminders of upcoming beam time. In each of these instances, the email messages are based on email templates that can be modified by user office staff within the *USO* system. The email server and account used by the system to send email notifications are configuration parameters, allowing the system to use a standalone email server or even an external cloud-based email service provider to send emails.

## 4. Impact on facility operations

The first integration of the *USO* at a large-scale research facility was accomplished at the CLS in 2018. As deployed, the system uses a central authentication system for authentication and a People Directory server as a source of user profile information. The People Directory is a separate external system where user permissions are assigned based on the completion of associated training through a separate but integrated training system. Roles and permissions are fetched and synchronized between the People Directory and the user portal through the synchronization APIs provided by the *USO* system.

To illustrate the flexibility of the *USO* system, the CLS operates 22 beamlines, each supporting a unique set of techniques and endstations, with varying access modes, including on-site experiments, remote operations and mail-in access. Proposals submitted by CLS users pass through one of three review tracks: general user (GU), macromolecular crystallography (MX) or rapid access (RA). All proposals are subject to a technical review by beamline staff to assess the suitability of the experiment to the requested beamline/instrument and the capability of the research team to complete the work. Proposals in the GU and MX tracks are reviewed for scientific merit by selected external reviewers, with separate peer-review committees comprising international experts for each track. Projects resulting from proposals passing through the GU and MX tracks are valid for two years. Proposals in the RA track are reviewed internally for scientific merit by CLS staff and are valid for six months after successful review. All aspects of the review process for each track are handled within the *USO* system.

At the CLS, all materials and samples associated with the project must pass a safety review before the experiment can proceed, and all the research team members participating during the session must have received the requisite permissions. Since 2018, these safety requirements have been easily satisfied through the *USO* software, eliminating the need for paper permits and manual verification of safety reviews and user training.

The CLS maintains its *USO* software instance through an internal software development group distinct from the original developers. This group also implements new site-specific features requested through the CLS user services office. The *USO* system has enabled the CLS user services office to evolve from a four-person team dedicated to spreadsheet manipulation, physical paper filing and manual process management to a lean, two-person team focusing on overall process improvements, user engagement, and supporting users and staff in their use of the *USO*. During this time, the system has managed over 5000 registrations, more than 4000 new proposals, more than 12000 scientific reviews, 3600 approved projects, 12500 experimental sessions, 4400 laboratory sessions and 7800 new publication entries, interacting with 5700 staff and users, and typically providing access for beam time to over 1000 users each year.

## 5. Adaptability

The *USO* software is open-source and can be adapted for similar large-scale facilities. Many core features are configurable and data-driven without any need to modify the source code. Source code changes can range in complexity from simply specifying different software settings within the configuration files to modifying the source code to add new functionality or changing workflow steps.

For example, other facilities can create different review tracks, review types and proposal forms with only data and configuration changes. Proposal and review forms can be modified using the built-in form designer, and associated review types can be created or modified to specify different proposal scoring schemes. As described in Section 2.1, interactions with external systems can also be adapted. Specifically, roles associated with reviews, beamlines and system administrators are configurable.

With open-source software, the possibilities of further adaptation and reuse are expansive. For example, some core modules, like the form designer or the publications management module, can be used alone without the rest of the system with minimal code changes. However, other modules, like the proposals and projects modules, are more integrated and therefore will require more code changes if used separately.

## 6. Conclusions

The *USO* software has been designed, implemented and integrated into facility operations at the CLS since 2018. The system has played an invaluable role since then. The publications management functionally was developed earlier and demonstrated such a significant improvement over the existing solution that it was deployed for use years before the rest of the system was fully developed.

Although initially developed for the CLS, the system was deliberately designed for adaptability to facilitate its use by other large-scale facilities. A different team of developers, working independently from the authors, successfully integrated and deployed the software at the CLS. Making the source code publicly available with an MIT-style open-source licence allows any large-scale facilities wishing to use the software to integrate and modify it to their needs freely.

The *USO* software has all the critical features required for operating a user facility, such as the CLS. However, we will consider adding more features to improve the system further. These include support for dual anonymous peer reviews, more configurable system parameters (cycle durations, shift start/end times *etc.*), integrating AI-based tools for reviewer assignment, a dynamic report designer and generator, and validity time limits for roles and permissions.

## Data Availability

The source code is publicly available at the GitHub repository https://github.com/michel4j/uso under an open-source license.
